# Elucidating the mechanisms by which acetyl tributyl citrate affects fracture healing: a comprehensive network toxicology study

**DOI:** 10.1186/s40360-026-01085-4

**Published:** 2026-02-02

**Authors:** Yining Chen, Caiyun Huang, Yuan Zhou, Zhongyuan Liu, Xialin Tang, Yanqiu Li, Chenkang Lu, Qiwang He

**Affiliations:** 1 Hubei University of Chinese Medicine, Wuhan, 430061 China; 2 Fujian University of Traditional Chinese Medicine, Fuzhou, 350108 China; 3 Department of Blood Transfusion, Xiangyang Central Hospital, Affiliated Hospital of Hubei University of Arts and Science, Xiangyang, 441021 China; 4 Traditional Chinese Medical Hospital of Changxing, Huzhou, 313100 China; 5 Capital Medical University, Beijing, 100069 China; 6 Hubei Provincial Hospital of Traditional Chinese Medicine, Affiliated Hospital of Hubei University of Chinese Medicine, Hubei Key Laboratory of Theory and Application Research of Liver and Kidney in Traditional Chinese Medicine, Hubei Province Academy of Traditional Chinese Medicine, Wuhan, 430061 China; 7 Hubei Shizhen Laboratory, Wuhan, 430061 China

**Keywords:** ATBC, Fracture healing, Network toxicology, Molecular dynamics simulation

## Abstract

**Background:**

In this study, we explored the potential risk effects of acetyl tributyl citrate (ATBC) on fracture healing through the method of network toxicology.

**Methods:**

ATBC-related targets were retrieved from the ChEMBL, Swiss Target Prediction, and STITCH databases, whereas fracture healing-related targets were obtained from the GeneCards, OMIM, and GEO databases. Core targets were identified through protein-protein interaction network construction using the STRING database and visualized using the Cytoscape software, followed by GO and KEGG enrichment analyses. Additionally, in-depth analyses, including immune infiltration profiling, gene set enrichment analysis, and ceRNA regulatory network construction, were conducted. Finally, molecular docking and dynamics simulations were performed to determine the binding efficiency and stability for the binding between ATBC and its core targets.

**Results:**

A total of 50 overlapping targets were identified, from which six core targets were selected. Using differential expression analysis, five core targets (HDAC2, HDAC3, KAT2B, SMARCA4, and TP53) were further refined. Enrichment analysis suggested potential mechanisms related to the thyroid hormone signaling pathway, the Notch signaling pathway, and the cell cycle. Molecular docking and dynamics simulations confirmed certain binding interactions between ATBC and the core targets.

**Conclusions:**

We provided an integrated network toxicology analysis framework coupled with a molecular dynamics evaluation framework to elucidate the underlying molecular mechanisms through which ATBC interferes with fracture healing, thereby providing novel therapeutic strategies for clinical intervention. However, the findings require further experimental validation and clinical confirmation to establish translational relevance.

**Trial registration:**

Not applicable.

## Background

Due to the mass production and extensive utilization of plastic products, environmental microplastic concentrations have increased considerably. Consequently, there is growing concern among researchers regarding the ecological and human health risks associated with prolonged exposure to microplastics. Recent investigations have revealed markedly elevated microplastic accumulation in human tissues, including detectable levels in cerebral structures [1]. As indispensable plastic softeners that are strongly positively correlated with plastic manufacturing outputs, plasticizers have drawn considerable attention. Acetyl tributyl citrate (ATBC), a representative plasticizer, is the preferred alternative to conventional phthalate-based counterparts because of its high thermal stability, chemical resistance, and unique solubility profile (water insoluble but miscible with organic solvents). Its applications span food-grade packaging materials, medical polyvinyl chloride devices, and toys. Although ATBC is classified as a safe additive, emerging toxicological studies have shown health risks associated with ATBC exposure, necessitating rigorous safety reevaluation.

Research on the environmental toxicological effects of ATBC is increasing. ATBC possesses high migration characteristics, which may increase its environmental concentrations [2]. Although ATBC exhibits low toxicity, prolonged exposure could exceed safety thresholds and induce cumulative toxic side effects in organisms [3]. Even low-dose ATBC can impair the proliferation and migration of mouse osteoblasts while disrupting balanced bone metabolism. The regulatory effects of ATBC on bone metabolism may be indirectly mediated through multiple complex signaling cascades [4]. Some studies have revealed that microplastics accumulate in human intervertebral discs, bones, and cartilage and that their infiltration influences inflammatory responses and bone morphogenetic factors. Given that ATBC is added as a plasticizer to plastic products, residual ATBC components may persist in microplastic particles formed during plastic degradation [5]. Considering the adverse effects of ATBC on bone metabolism, we hypothesized that ATBC might disrupt the osteogenic-osteoclastic balance, thereby contributing to impaired fracture healing-related conditions, including nonunion and delayed fracture healing.

Fracture healing is a complex biological process that can be broadly categorized into two types, including primary bone healing and secondary fracture healing [6], and three phases: the inflammatory phase, soft and hard callus formation, and the remodeling phase [7]. It involves the coordination, collaboration, and interaction of multiple cells and molecular signaling pathways [8]. Under normal conditions, damaged bone post-fracture can regenerate to restore its pre-injury cellular composition, structure, and biomechanical functionality [6]. However, factors such as diabetes, aging, cellular senescence, and reduced macrophage populations [8, 9] may impair fracture healing, leading to complications such as nonunion, delayed union, and bone defects. About 10% of fractures fail to heal properly, causing a significant reduction in the quality of life of patients and imposing a substantial economic burden on healthcare systems [10]. Therefore, identifying novel potential risk factors for impaired fracture healing remains a critical unmet clinical need in orthopedics. Some studies have demonstrated that nanoplastics disrupt the bone microenvironment by suppressing osteoblast migration, promoting osteoclastogenesis, and increasing the levels of inflammatory factors [11]. As plasticizers are a crucial component of plastics, the lack of research on the role of ATBC in fracture healing highlights the importance of investigating its risk mechanisms. Such studies would not only address a research gap but also align with urgent clinical demands.

In recent decades, plastic waste has increased exponentially. As plastic debris is released into the environment, it may be deposited in the human body through multiple pathways [12] and exert harmful effects on their health [13]. However, research on the toxicological profiles of such plastic pollutants is largely inadequate [14]. Recent network toxicology studies have overcome the limitations of traditional toxicological methods using multiple databases to construct relationship networks among compounds, toxicity, and targets, enabling a comprehensive and effective assessment of the health risks posed by the ever-increasing environmental pollutants to humans [15, 16]. With advantages such as high accuracy, operational convenience, broad applicability, and strong potential for deep data mining, network toxicology provides necessary technical support for predicting chemical toxicity, deciphering molecular mechanisms, and screening hazardous substances [17, 18]. Although ATBC is a safer alternative plasticizer, prolonged human exposure to high concentrations of ATBC needs to be investigated for its potential harmful effects due to bioaccumulation. The mechanisms underlying the toxicological effects of ATBC on fracture healing are not clear. Therefore, in this study, we implemented network toxicology to assess the risk mechanisms of ATBC in fracture healing, offering novel insights into and theoretical support for understanding impaired fracture healing.

## Materials and methods

**Fig. 1:**
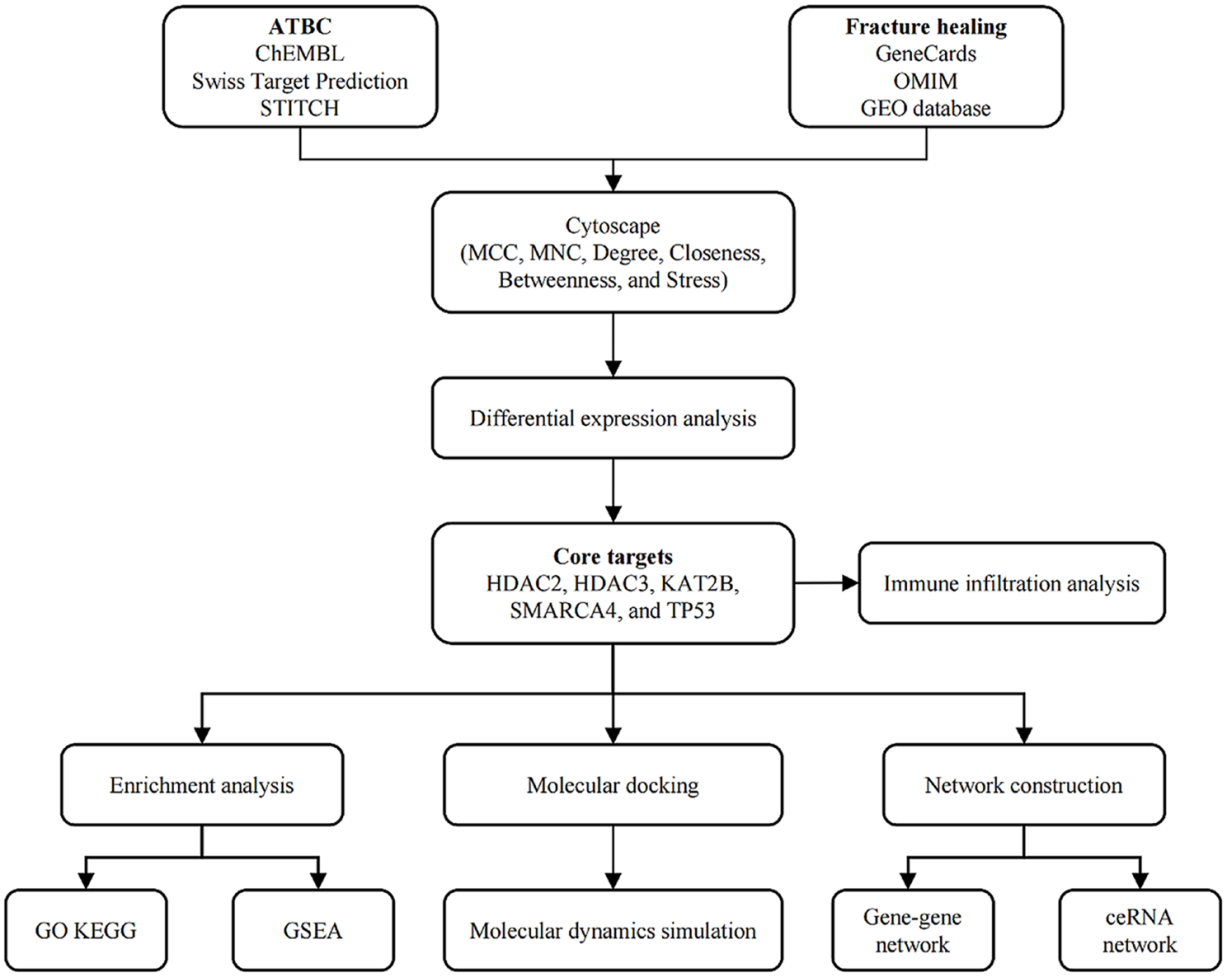
The flowchart of this study

The overall research design flowchart is shown in Fig. 1.

### Collection of ATBC targets

By querying “acetyl tributyl citrate” in the PubChem database (https://pubchem.ncbi.nlm.nih.gov/), the standard structure and SMILES symbol of ATBC were identified (PubChem release 2021.10.14). Based on these results, potential ATBC targets were retrieved from the ChEMBL database (https://www.ebi.ac.uk/chembl/) using the keyword “acetyl tributyl citrate”, with species restricted to “*Homo sapiens*”. The SMILES symbol ATBC was uploaded to the Swiss Target Prediction database (http://www.swisstargetprediction.ch/) and the STITCH database (http://stitch.embl.de/) to identify overlooked potential targets (the species is limited to “*Homo sapiens*”). Next, targets from different sources were integrated, duplicates were removed, and structural consistency was confirmed. Then, the UniProt database (https://www.uniprot.org/) was used to standardize the nomenclature of the obtained targets. Finally, a comprehensive ATBC target library encompassing all consolidated and validated targets was established [4].

### Selection of the fracture healing-related target network

Fracture healing-related targets were retrieved from the GeneCards (https://www.genecards.org/) and OMIM (https://www.omim.org/) databases. The keyword “fracture healing” was searched in the GEO database (https://www.ncbi.nlm.nih.govgeo/) to obtain fracture healing-related datasets. The GSE93215 dataset was downloaded from the GPL6244 [HuGene-1_0-st] Affymetrix Human Gene 1.0 ST Array [transcript (gene) version] platform, which contains peripheral blood microarray data from 19 acute fracture patients and 9 healthy volunteer controls [19].

The R language “limma” package (v 3.58.1) was used to analyze differentially expressed genes (DEGs) in the GSE93215 dataset, with a significance threshold of *P* < 0.05. The data from the GeneCards and OMIM databases were subsequently merged, duplicates were removed (union operation), and the results were combined with the GSE93215 dataset while overlapping entries were retained (intersection operation). Finally, a specialized fracture healing target library was established.

The “VennDiagram” package (v 1.7.3) was used to identify common targets between ATBC and fracture healing, whereas the “ggplot2” package (v 3.4.4) was used to visualize the results. The overlapping targets were designated as potential targets through which ATBC may influence fracture healing.

### Construction of the protein-protein interaction (PPI) network and core target selection

The potential target genes through which ATBC influences fracture healing were input into the STRING database (https://string-db.org/), with the species restricted to “*Homo sapiens*” and a “minimum required interaction score” set to “medium confidence > 0.4” for analysis. The data generated using the STRING database were subsequently imported into the Cytoscape software for visualization. The cytoHubba plugin was used to identify core targets using six algorithms: MCC, MNC, Degree, Closeness, Betweenness, and Stress. Subsequently, an UpSet plot was generated to determine the intersection of the top 10 core targets ranked by the six algorithms. Furthermore, the expression levels of core targets obtained from the GSE93215 dataset were analyzed for differential expression between the disease group and the control group for preliminary validation.

### Functional and pathway enrichment analysis of core targets

To elucidate the potential mechanisms underlying the influence of ATBC on fracture healing, the “clusterProfiler” package (v 4.4.4) was used to perform Gene Ontology (GO) and Kyoto Encyclopedia of Genes and Genomes (KEGG) enrichment analyses on the core targets. A comprehensive GO analysis was conducted, encompassing biological process (BP), cellular component (CC), and molecular function (MF) terms. Next, KEGG analysis was performed to identify pathways associated with the effect of ATBC on fracture healing. Finally, the results were visualized as bar plots and bubble plots using the “ggplot2” package, with a significance threshold of adj. *P* < 0.05.

### Immune infiltration analysis

The single-sample gene set enrichment analysis (ssGSEA) from the “GSVA” package (v 1.44.5) was applied to calculate the infiltration levels of 24 immune cell types based on gene expression profiles from published immune cell gene sets. The associations between the core targets and these immune cells were further assessed.

### GSEA for core targets

Gene set enrichment analysis (GSEA) was performed using the “clusterProfiler” package to investigate the biological functions of the core targets in detail, and the “ggplot2” package was used for data visualization.

### Network construction based on core targets

Potential core targets-associated genes were retrieved using the GeneMANIA database (https://genemania.org/) to construct a gene-gene interaction network [20]. The starBase database (https://rnasysu.com/encori/) was used to predict the interactions of miRNAs and lncRNAs with core targets (set “CLIP-Data ≥ 4”), facilitating the construction of a competing endogenous RNA (ceRNA) regulatory network involving mRNA-miRNA-lncRNA interactions.

### Molecular docking validation

In this study, we used the following technical workflow for molecular interaction analysis. First, core protein structures were retrieved from the RCSB Protein Data Bank (https://www.rcsb.org/), whereas the molecular structure of ATBC was downloaded from the PubChem compound database. The PyMOL software molecular visualization platform was used to optimize the protein structures, which were ultimately exported in the standardized PDB format. Subsequently, automated molecular docking simulations were conducted using the deep learning-based CB-Dock2 online docking platform (https://cadd.labshare.cn/cb-dock2/php/index.php/), which autonomously performs critical steps such as binding cavity identification and conformational sampling. Finally, the multi-structure alignment function of PyMOL was used to systematically analyze the three-dimensional binding characteristics between ATBC and the core targets, while its visualization module was used to generate professional-grade molecular interaction diagrams.

### Molecular dynamics simulation

We investigated the dynamic behavior of biomolecular systems by integrating multiscale molecular simulation techniques. The workflow was implemented as follows. First, the target small molecule was structurally optimized using the AmberTools22 software package. The molecular system was parameterized using the GAFF general force field, and hydrogen atom optimization was performed along with restrained electrostatic potential (RESP) charge fitting using the Gaussian16W quantum chemistry platform. The derived electrostatic parameters were integrated into the molecular dynamics topology files. Molecular dynamics simulations were conducted using the Gromacs (v 2022.3) software [21], with the amber99sb-ildn force field selected to describe protein-ligand interactions. The TIP3P water model was used to construct the solvation environment. Charge neutrality was achieved by adding sodium ions. The simulations were strictly maintained under isothermal-isobaric conditions (300 K, 1 bar) following a stepwise equilibration protocol: (1) energy minimization was achieved using the steepest descent algorithm; (2) two-phase equilibration—constant number of particles, volume, and temperature (NVT) and constant pressure, constant-temperature (NPT)—each involved 100,000 steps (coupling constant 0.1 ps, total duration 100 ps); and (3) a final production simulation of 100 ns (5,000,000 steps, 2 fs step size) was performed. Trajectory analysis utilized integrated analytical modules to systematically compute key biophysical parameters: (1) protein conformational stability metrics (RMSD: root mean square deviation; RMSF: root mean square fluctuation), (2) structural compactness via radius of gyration (Rg), (3) solvation effects measured by solvent-accessible surface area (SASA), (4) thermodynamic characteristics through molecular mechanics/generalized Born surface area (MM/GBSA) binding free energy calculations, and (5) conformational landscape features analyzed using free energy landscape (FEL).

## Results

### Identification of ATBC targets influencing fracture healing

**Fig. 2:**
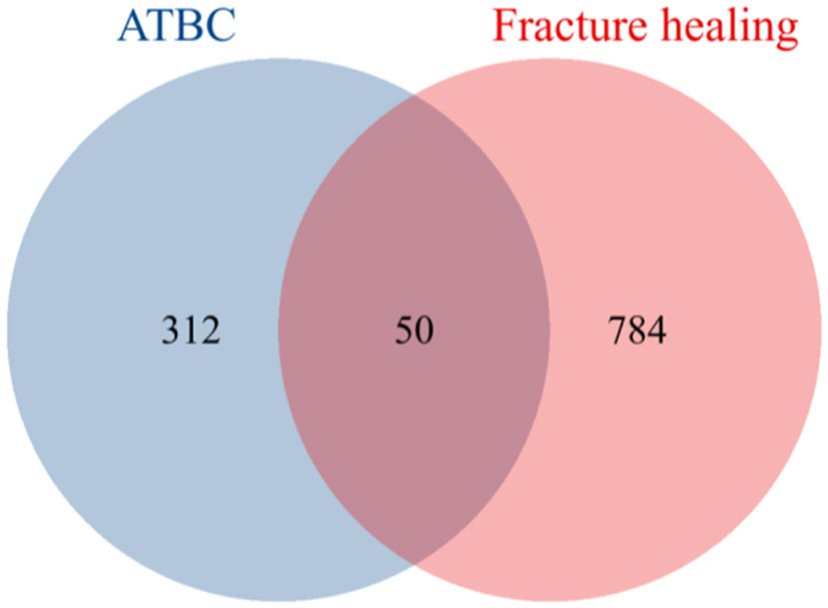
Venn diagram of the intersection between ATBC and fracture healing targets

By integrating the ChEMBL, SwissTargetPrediction, and STITCH databases, we identified 362 ATBC-related targets (union of three databases). A total of 3,730 fracture healing-related targets were obtained from the GeneCards and OMIM databases (union of two databases), and 4,903 DEGs were screened from the GSE93215 dataset. Integrating the results from the databases and the dataset yielded 834 fracture healing-related targets (intersection of databases and dataset). Subsequently, 50 overlapping targets shared between ATBC and fracture healing were identified (Fig. 2), which represent potential candidates mediating the effect of ATBC on fracture healing.

### PPI network construction and core target identification

**Fig. 3:**
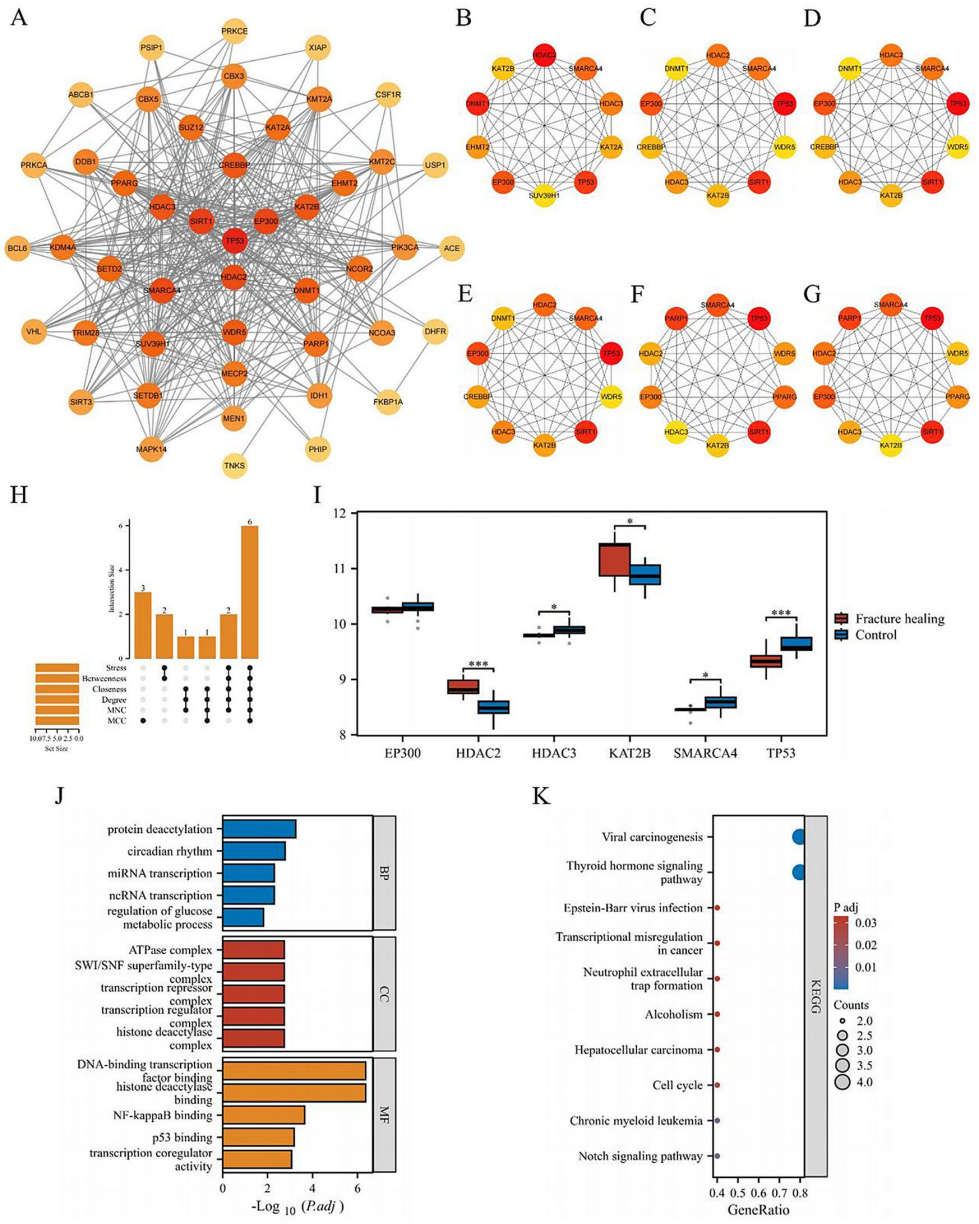
Screening and enrichment analysis of core targets. (**A**) PPI network of shared potential targets. Core target networks identified by (**B**) MCC, (**C**) MNC, (**D**) Degree, (**E**) Closeness, (**F**) Betweenness, and (**G**) Stress algorithms. (**H**) The UpSet plot shows intersecting core targets. (**I**) Differential expression analysis. (**J**) GO analysis of core targets. (**K**) KEGG analysis of core targets

A PPI network comprising 47 nodes and 382 edges was generated using the STRING database (Fig. 3A). The cytoHubba plugin was used to rank the top 10 potential targets based on six algorithms, including MCC (Fig. 3B), MNC (Fig. 3C), Degree (Fig. 3D), Closeness (Fig. 3E), Betweenness (Fig. 3F), and Stress (Fig. 3G). Six overlapping targets (EP300, HDAC2, HDAC3, KAT2B, SMARCA4, and TP53) were identified across all six algorithms (Fig. 3H). Next, five core targets were screened through differential expression analysis (Fig. 3I).

### GO and KEGG enrichment analyses of core targets

Enrichment analysis of the five core targets yielded 496 statistically significant GO terms, including 403 BP terms, 31 CC terms, and 62 MF terms. As shown in Fig. 3J, the targets were enriched predominantly in BP categories such as protein deacetylation, ncRNA transcription, and miRNA transcription. For CC categories, enrichment was observed in histone deacetylase complex, transcription regulator complex, and ATPase complex. In the MF categories, the targets were enriched mainly in histone deacetylase binding, NF-κB binding, and p53 binding.

Additionally, 11 statistically significant KEGG pathways were enriched. The core targets linking ATBC and fracture healing were associated primarily with signaling pathways such as the thyroid hormone signaling pathway, the Notch signaling pathway, the cell cycle, and the neutrophil extracellular trap formation (Fig. 3K).

### Immune infiltration analysis

**Fig. 4:**
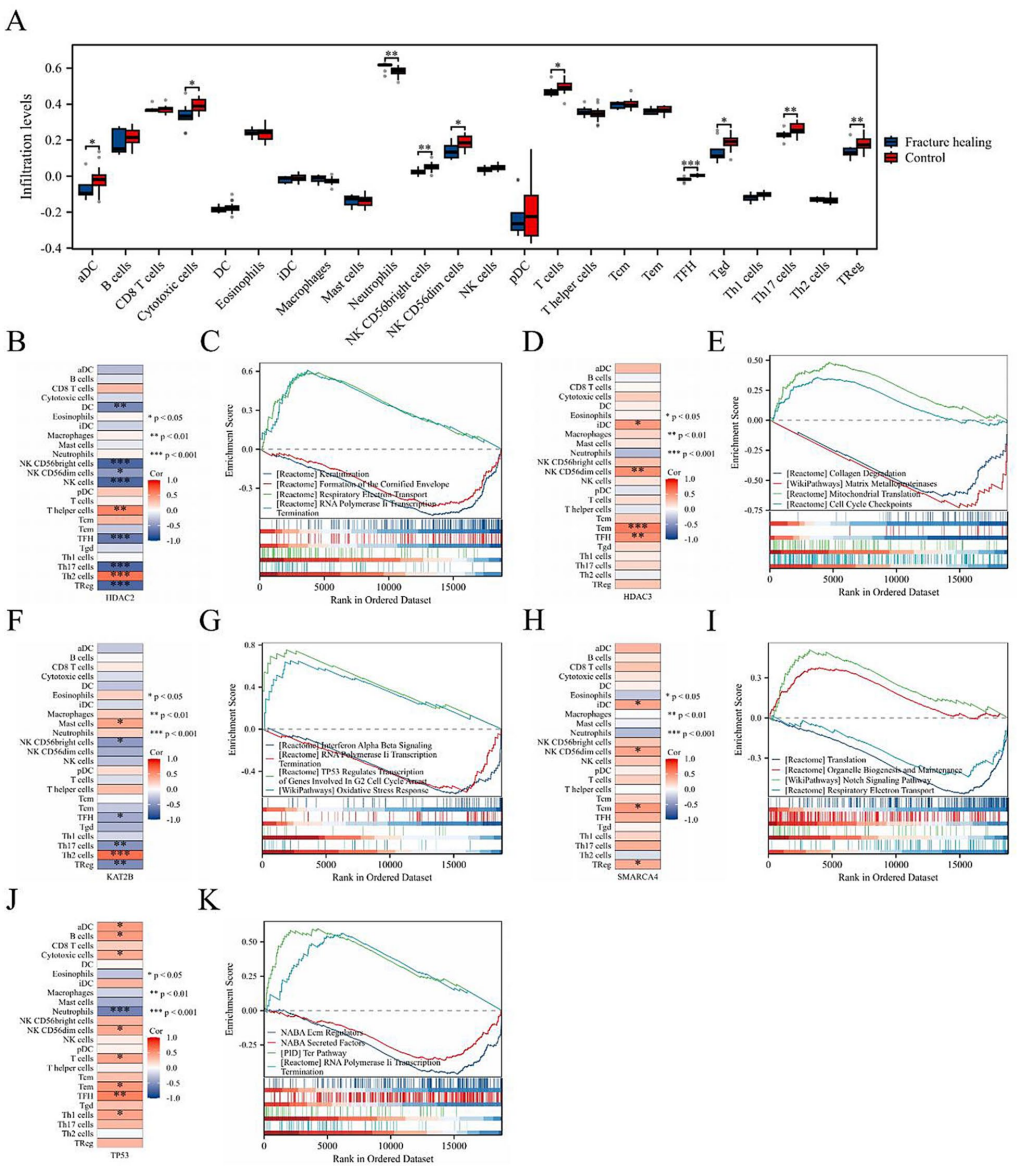
Immune infiltration and GSEA analyses. (**A**) Group comparison plots of 24 immune cell types between the fracture healing and control groups. Heatmaps showing immune cell infiltration correlations for HDAC2 (**B**), HDAC3 (**D**), KAT2B (**F**), SMARCA4 (**H**), and TP53 (**J**). GSEA enrichment plots for HDAC2 (**C**), HDAC3 (**E**), KAT2B (**G**), SMARCA4 (**I**), and TP53 (**K**)

The ssGSEA algorithm was used to assess immune cell infiltration between the disease and control groups in the fracture healing dataset. The results revealed higher neutrophil levels in the disease group, whereas a decrease in infiltration was observed for aDCs, cytotoxic cells, natural killer (NK) CD56 bright cells, NK CD56 dim cells, T cells, TFH, Tgd, Th17 cells, and regulatory T cells (Tregs) (Fig. 4A). Additionally, the five core targets exhibited significant correlations with multiple immune cell types (Figs. 4B, D, F, H and J).

### GSEA enrichment analysis of core targets

We performed GSEA on the five core targets to evaluate their roles in the initiation and progression of fracture healing. The results revealed that HDAC2 was enriched primarily in pathways such as RNA polymerase II transcription termination and keratinization envelope formation (Fig. 4C). HDAC3 was significantly enriched in collagen degradation and matrix metalloproteinases (Fig. 4E). KAT2B was significantly enriched in RNA polymerase II transcription termination and oxidative stress response (Fig. 4G). SMARCA4 was significantly enriched in Notch signaling pathway and respiratory electron transport (Fig. 4I). TP53 was significantly enriched in T-cell receptor pathway and secretory factor-related processes (Fig. 4K).

### Gene-gene interaction network

**Fig. 5:**
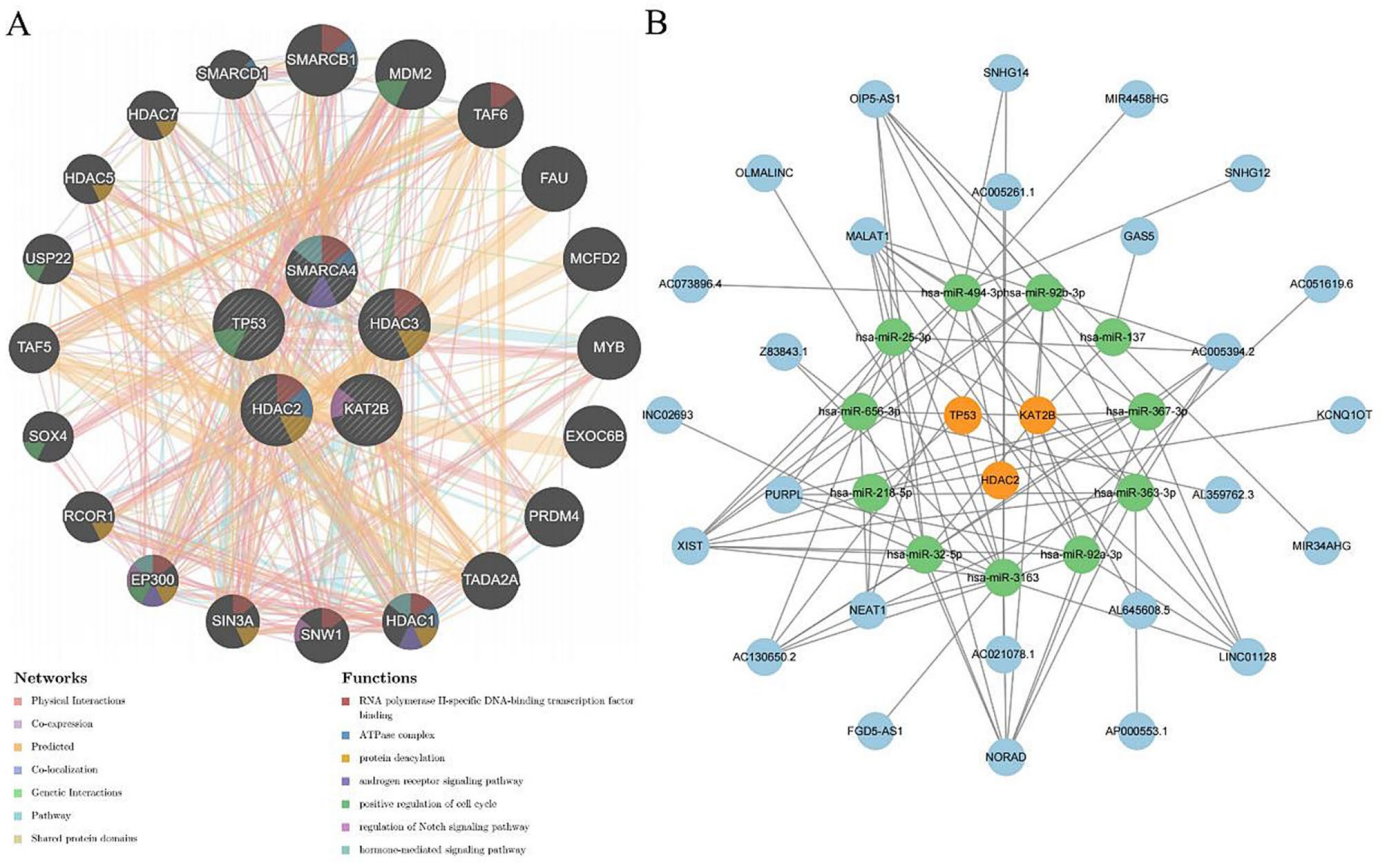
Construction of interaction network. (**A**) Gene-gene interaction network. (**B**) ceRNA regulatory network

The gene-gene interaction network of the core targets is illustrated in Fig. 5A, in which central hub nodes symbolize these genes, surrounded by 20 nodes (SMARCB1, MDM2, TAF6, FAU, MCFD2, MYB, EXOC6B, PRDM4, TADA2A, HDAC1, SNW1, SIN3A, EP300, RCOR1, SOX4, TAF5, USP22, HDAC5, HDAC7, and SMARCD1), representing genes significantly associated with them. Additionally, the network highlighted the top seven related functional terms, such as the RNA polymerase II-specific DNA-binding transcription factor binding, the ATPase complex, and the androgen receptor signaling pathway.

### ceRNA network

The mRNA-miRNA-lncRNA ceRNA regulatory network of the biomarkers is shown in Fig. 5B. Through miRNA prediction, 11 miRNAs with potential associations with three targets were identified. Subsequently, lncRNA prediction using the starBase database revealed 26 lncRNAs potentially interacting with these miRNAs.

### Molecular docking validation

**Fig. 6:**
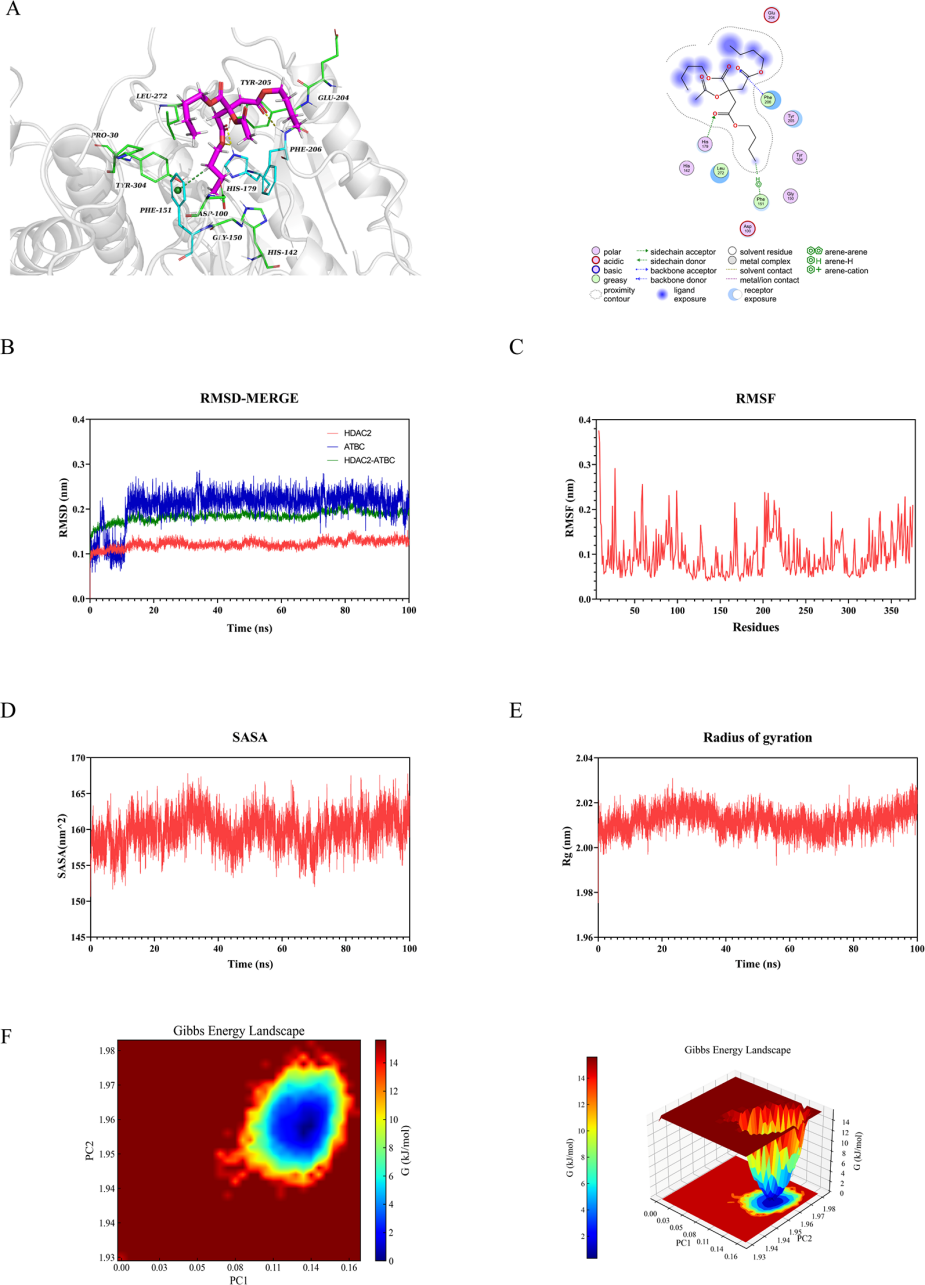
Validation of HDAC2 molecular docking and molecular dynamics simulations. (**A**) Molecular docking validation of HDAC2. (**B**) RMSD values of the HDAC2-ATBC complex over time. (**C**) RMSF values of backbone atoms in the HDAC2-ATBC complex over time. (**D**) SASA values of the HDAC2-ATBC complex over time. (**E**) Rg values of the HDAC2-ATBC complex over time. (**F**) The free energy landscape of HDAC2-ATBC complex

**Fig. 7:**
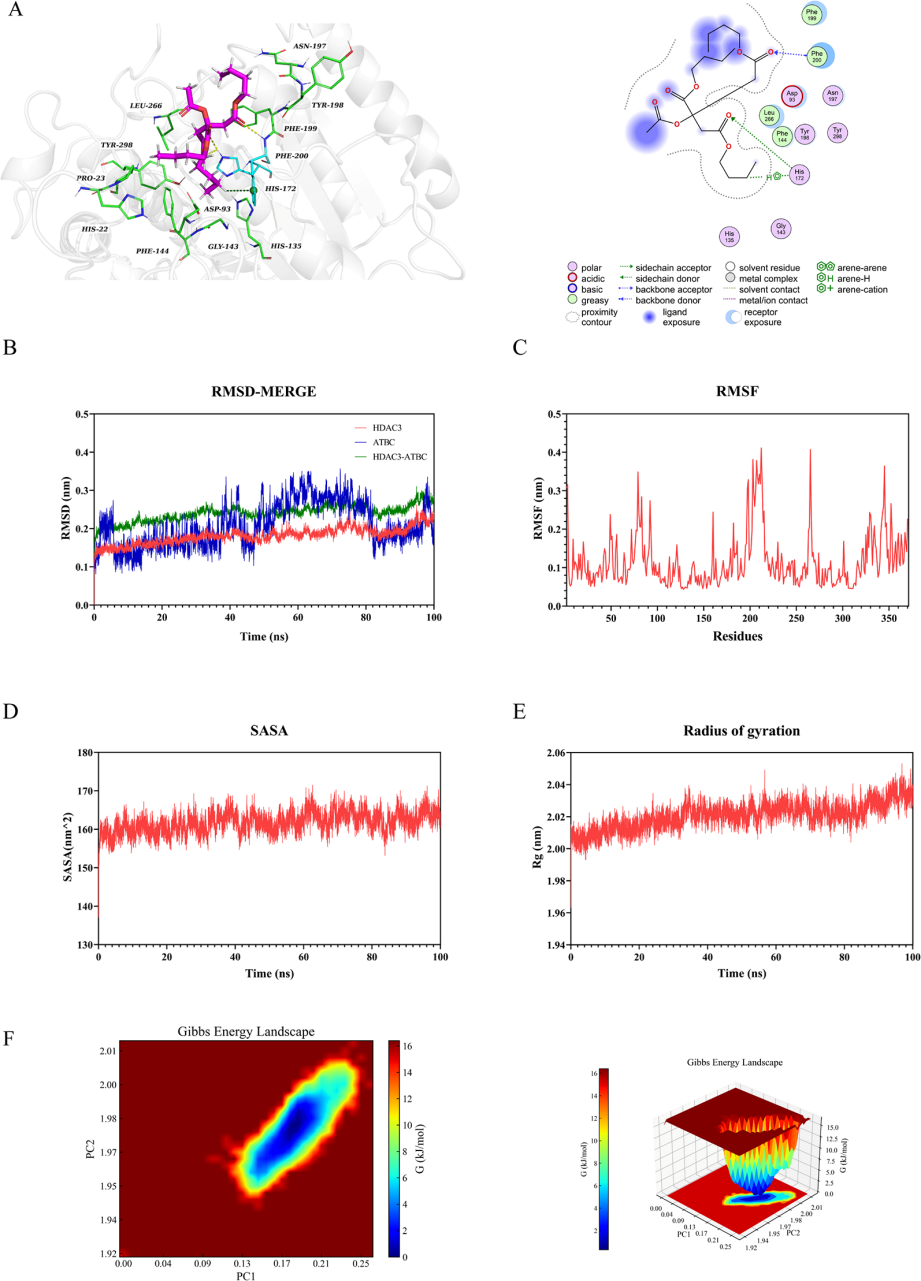
Validation of HDAC3 molecular docking and molecular dynamics simulations. (**A**) Molecular docking validation of HDAC3. (**B**) RMSD values of the HDAC3-ATBC complex over time. (**C**) RMSF values of backbone atoms in the HDAC3-ATBC complex over time. (**D**) SASA values of the HDAC3-ATBC complex over time. (**E**) Rg values of the HDAC3-ATBC complex over time. (**F**) The free energy landscape of HDAC3-ATBC complex

**Fig. 8:**
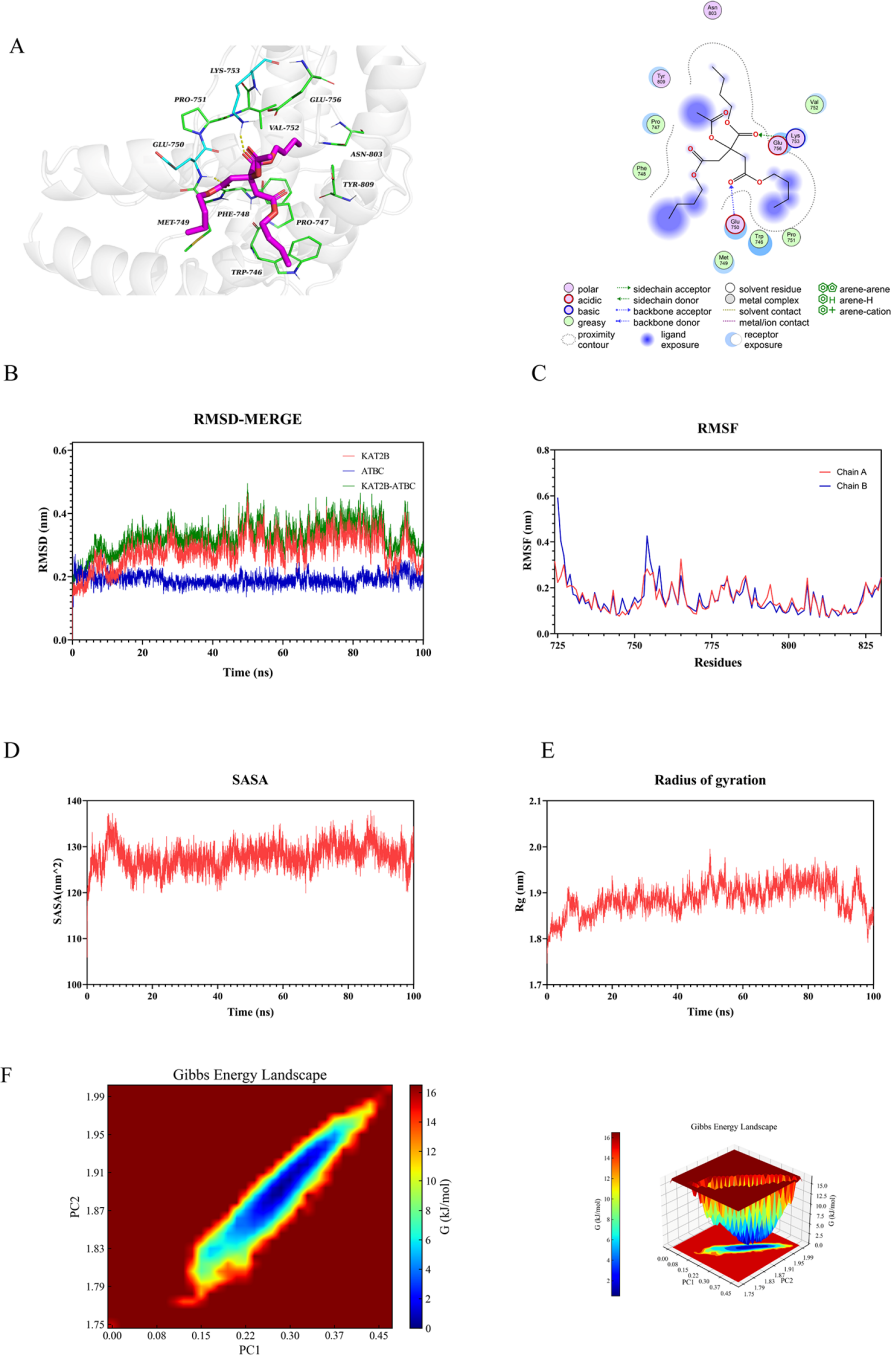
Validation of KAT2B molecular docking and molecular dynamics simulations. (**A**) Molecular docking validation of KAT2B. (**B**) RMSD values of the KAT2B-ATBC complex over time. (**C**) RMSF values of backbone atoms in the KAT2B-ATBC complex over time. (**D**) SASA values of the KAT2B-ATBC complex over time. (**E**) Rg values of the KAT2B-ATBC complex over time. (**F**) The free energy landscape of KAT2B-ATBC complex

**Fig. 9:**
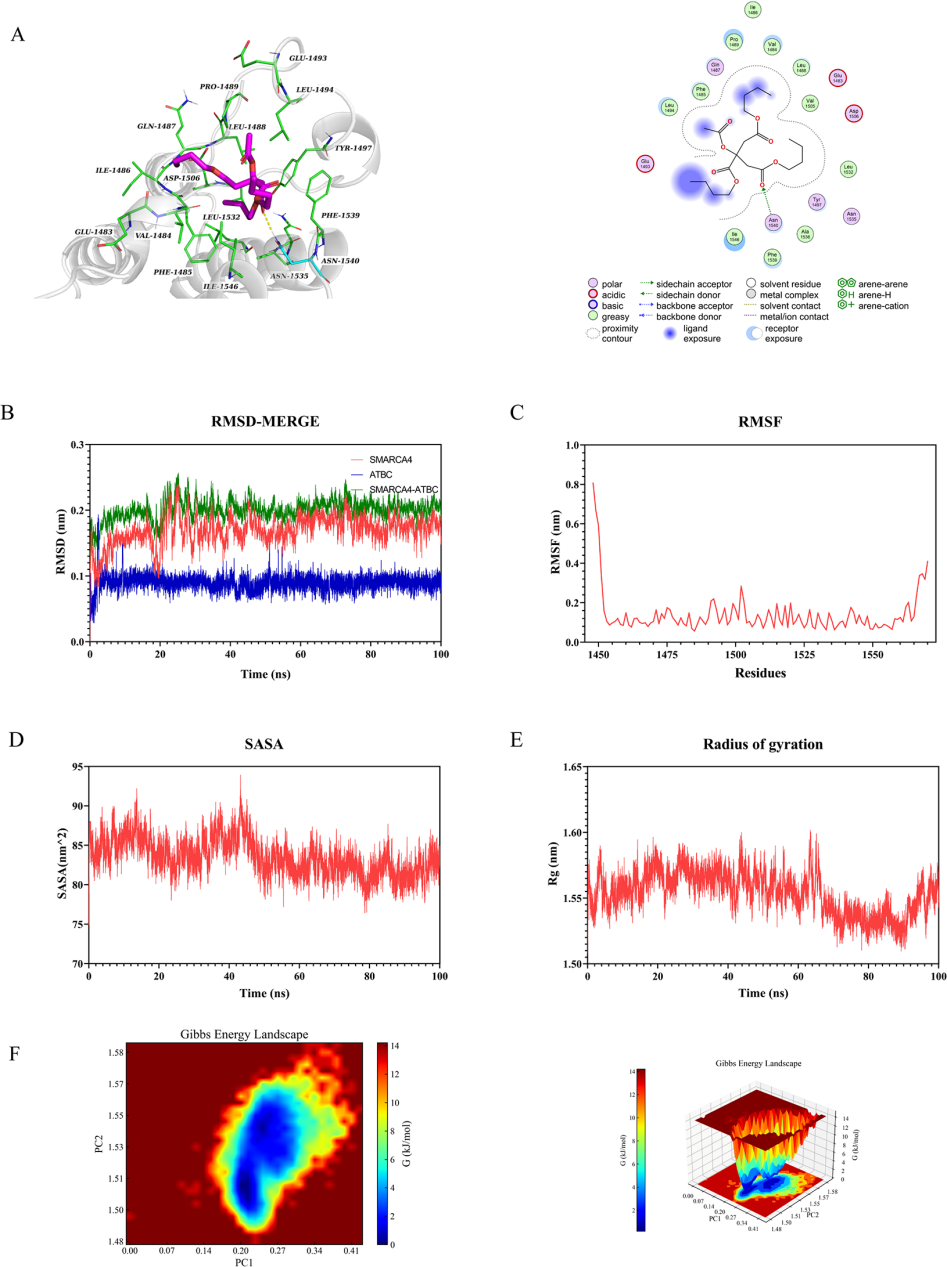
Validation of SMARCA4 molecular docking and molecular dynamics simulations. (**A**) Molecular docking validation of SMARCA4. (**B**) RMSD values of the SMARCA4-ATBC complex over time. (**C**) RMSF values of backbone atoms in the SMARCA4-ATBC complex over time. (**D**) SASA values of the SMARCA4-ATBC complex over time. (**E**) Rg values of the SMARCA4-ATBC complex over time. (**F**) The free energy landscape of SMARCA4-ATBC complex

**Fig. 10:**
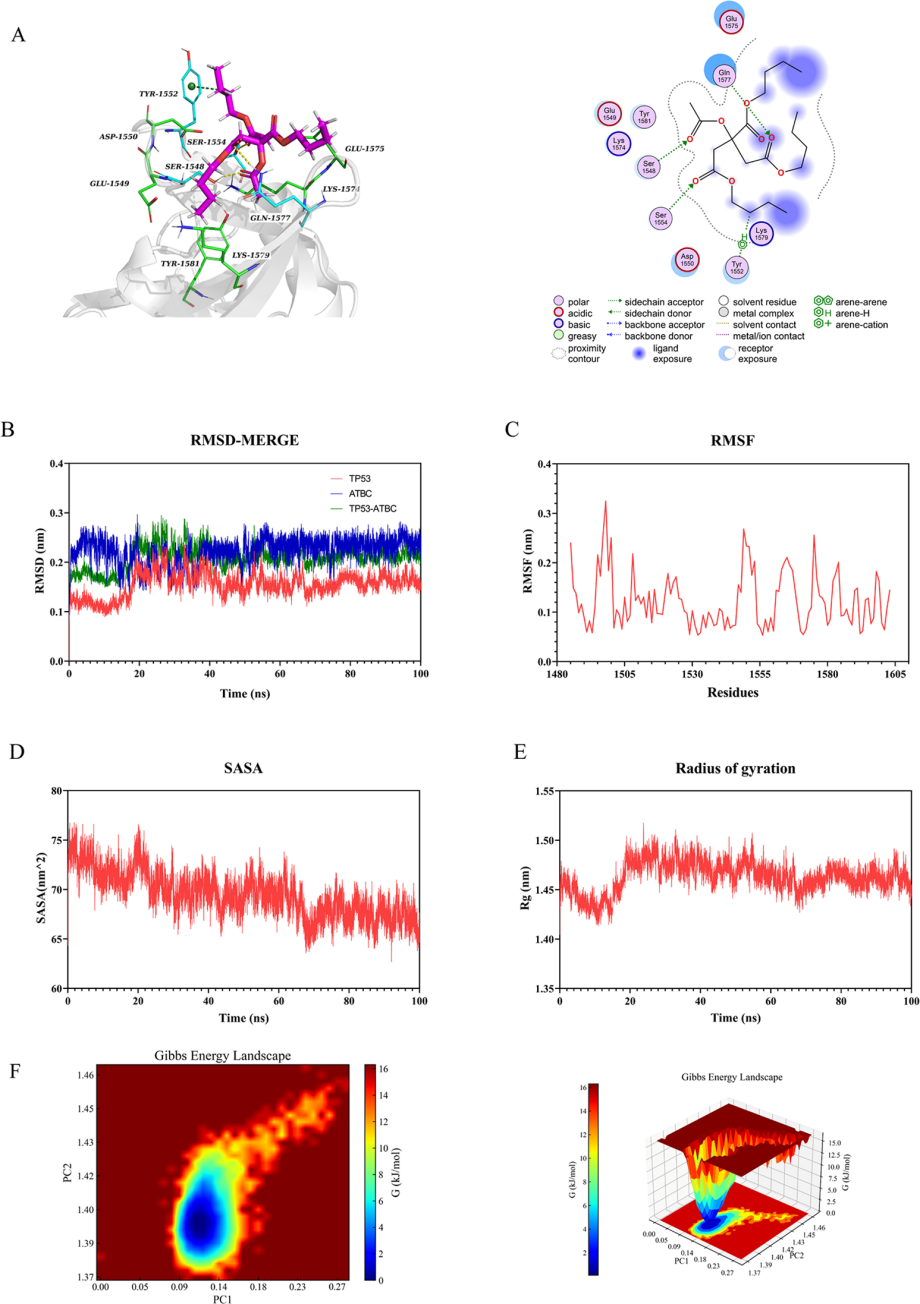
Validation of TP53 molecular docking and molecular dynamics simulations. (**A**) Molecular docking validation of TP53. (**B**) RMSD values of the TP53-ATBC complex over time. (**C**) RMSF values of backbone atoms in the TP53-ATBC complex over time. (**D**) SASA values of the TP53-ATBC complex over time. (**E**) Rg values of the TP53-ATBC complex over time. (**F**) The free energy landscape of TP53-ATBC complex

Molecular docking was performed to investigate the interactions between ATBC and five proteins. The findings revealed that the binding energy of the five target proteins ranged from − 4.4 to − 5.8 kcal/mol: HDAC2 (–4.8 kcal/mol, Fig. 6A), HDAC3 (–4.9 kcal/mol, Fig. 7A), KAT2B (–5.8 kcal/mol, Fig. 8A), SMARCA4 (–4.4 kcal/mol, Fig. 9A), and TP53 (–5.2 kcal/mol, Fig. 10A). All core targets exhibited favorable binding efficiency and stability with ATBC, as indicated by binding energies < − 4.25 kcal/mol. − 4.25 kcal/mol is widely recognized as the critical threshold for certain binding activity, used to screen molecules with potential binding capabilities [22]. This finding indicates spontaneous binding between ATBC and five core targets [23], which further supports their critical roles in the molecular mechanisms underlying the effect of ATBC on fracture healing.

### Molecular dynamics simulation

To confirm the molecular docking results for five protein-ligand complexes, molecular dynamics simulations were conducted. The RMSD curves of the five protein-ligand complexes stabilized after 20 ns (Figs. 6B, 7B, 8B, 9B and 10B), and remained relatively stable thereafter, respectively. RMSF analysis identified key flexible regions in the proteins, and the RMSF values of most residues fluctuated in the range of 0.10–0.20 nm (Figs. 6C, 7C, 8C, 9C and 10C), indicating structural stability. The SASA values remained stable at about 165 nm^2^ (Fig. 6D), 161 nm^2^ (Fig. 7D), 127 nm^2^ (Fig. 8D), 83nm^2^ (Fig. 9D), and 72 nm^2^ (Fig. 10D). The Rg for five complexes also stabilized, with average values of 2.01 nm (Fig. 6E), 2.02 nm (Fig. 7E), 1.89 nm (Fig. 8E), 1.55 nm (Fig. 9E), and 1.44 nm (Fig. 10E).

Further, the binding free energy was calculated using the MM/GBSA method to quantify the binding affinity. As shown in Table 1, the average free energies of HDAC2, HDAC3, KAT2B, SMARCA4, and TP53 binding to ATBC were − 24.89 ± 1.49 kcal/mol, − 21.84 ± 1.30 kcal/mol, − 21.08 ± 2.29 kcal/mol, − 30.25 ± 1.27 kcal/mol, and − 19.86 ± 1.73 kcal/mol. These results indicated strong binding affinities between ATBC and five core targets, driven primarily by hydrophobic effects and electrostatic interactions. The thermodynamically favorable binding further supported the molecular docking findings, confirming the reliability of the interactions between ATBC and the five core targets in impairing fracture healing.

**Table 1:** MM/GBSA parameters for the five protein-ligand complexes

Energy	HDAC2	HDAC3	KAT2B	SMARCA4	TP53
ΔVDWAALS	-31.84 ± 1.33	-28.85 ± 0.03	-31.49 ± 2.12	-38.86 ± 0.08	-29.08 ± 0.62
ΔE_elec_	-13.29 ± 0.10	-8.93 ± 1.14	-15.30 ± 0.76	-3.45 ± 1.25	-17.09 ± 0.02
ΔE_GB_	25.05 ± 0.68	20.15 ± 0.63	30.31 ± 0.40	17.54 ± 0.18	31.29 ± 1.61
ΔE_surf_	-4.81 ± 0.06	-4.21 ± 0.01	-4.60 ± 0.10	-5.47 ± 0.04	-4.99 ± 0.06
ΔG_gas_	-45.13 ± 1.33	-37.78 ± 1.14	-46.79 ± 2.25	-42.31 ± 1.25	-46.17 ± 0.62
ΔG_solvation_	20.24 ± 0.68	15.94 ± 0.63	25.71 ± 0.41	12.06 ± 0.18	26.30 ± 1.61
ΔTotal	-24.89 ± 1.49	-21.84 ± 1.30	-21.08 ± 2.29	-30.25 ± 1.27	-19.86 ± 1.73

ΔVDWAALS: van der Waals energy; ΔE_elec_: electrostatic energy; ΔE_GB_: polar solvation energy; ΔE_surf_: nonpolar solvation energy; ΔG_gas_: molecular mechanical term energy (meteorological chemical energy) = ΔVDWAALS + ΔE_elec_; ΔG_solvation_: solvation energy = ΔE_GB_ + ΔE_surf_; ΔTOTAL: comprehensive total energy = ΔG_gas_ + ΔG_solvation_

We conducted a multidimensional conformational space analysis to systematically elucidate the dynamic stability characteristics of the complex. Based on principal component analysis (PCA) of the RMSD and Rg, the principal component vectors PC1 and PC2 were selected as the axes of the two-dimensional conformational space. These values were combined with Gibbs free energy values to construct a three-dimensional FEL (Figs. 6F, 7F, 8F, 9F and 10F). Thermodynamic analysis revealed that the dark blue energy basins corresponded to the dominant conformational states of the complex, with energy characteristics exhibiting a typical unimodal distribution pattern. The free energy surface showed a smooth gradient and steep potential wells in the lowest-energy regions. While unstable protein-ligand interactions often lead to polymorphic energy basins with increased surface roughness in free energy topography, all systems in this study showed singular low-energy aggregation zones in their FELs. This finding indicates highly convergent conformational dynamics across the molecular dynamics trajectories. Our results aligned with the narrow fluctuation range of the RMSD trajectories, collectively confirming the structural stability of the complex under physiological conditions. This thermodynamic stability may increase the in vivo residence time of the five complexes, allowing sustained modulation of target protein function to influence bone metabolic homeostasis. These findings provide important theoretical insights into the molecular pathological mechanism by which ATBC affects fracture healing.

## Discussion

Impaired fracture healing, a common complication following post-traumatic orthopedic surgery, can significantly reduce the quality of life of patients, with severe cases potentially resulting in permanent functional disabilities [24]. However, conventional diagnostic and therapeutic approaches for impaired fracture healing have notable limitations. Traditional X-ray evaluation techniques suffer from a temporal discrepancy between radiographic manifestations and biological healing progression [25]. Additionally, the available clinical nonunion prediction scoring systems, which predominantly rely on clinical and imaging parameters, may overlook critical biomarkers or dynamic pathophysiological factors [26]. To address these challenges, in this study, we systematically identified 50 candidate targets associated with ATBC-mediated modulation of fracture healing by integrating multi-source databases, including ChEMBL, GeneCards, and GEO. A PPI network was constructed using the STRING database, and topological analysis conducted using the Cytoscape software revealed five core regulatory targets. This is the first study to elucidate the molecular mechanism by which ATBC influences fracture healing, providing a novel clinical perspectives for managing impaired fracture healing.

Histone deacetylase 2 (HDAC2), a key member of the class I histone deacetylase family, primarily regulates chromatin structure and gene expression by removing acetyl groups from lysine residues on the N-terminal tails of histones. Several studies have reported the critical role of HDAC2 in bone repair. For example, HDAC2 can suppress inflammatory responses by regulating FOXO1, thereby indirectly influencing skeletal health [27]. Additionally, HDAC2 plays a key role as an enzyme in osteoblast differentiation. Silencing HDAC2 increases the expression of osteopontin (OPN) and bone sialoprotein (BSP) while downregulating osteocalcin (OC) mRNA levels [28]. HDAC2 inhibitors promote histone deacetylation, altering chromatin structure to modulate the expression of specific genes, such as enhancing the expression of osteogenic markers such as Runx2 and OCN, which further drives osteogenic differentiation. Additionally, HDAC2 inhibitors facilitate the osteogenic differentiation of bone marrow mesenchymal stem cells (BMSCs) [29]. As osteogenic differentiation is a critical phase in fracture healing, HDAC2 probably plays a central role in this BP.

HDAC3 is a zinc-dependent histone deacetylase. Unlike other HDACs, it shuttles between the nucleus and cytoplasm and possesses dual enzymatic and non-enzymatic functions [30]. In the skeletal system, HDAC3 is highly expressed in chondrocytes (at resting and prehypertrophic phases), osteoblasts, and osteocytes [30, 31]. Furthermore, HDAC3 serves as a key negative regulator of osteoblast differentiation. It can bind to Runx2, the master transcription factor of osteoblasts, to inhibit its transcriptional activity, thereby regulating the promoter activity of OC and ultimately affecting the process of bone mineralization [32]. Meanwhile, relevant studies have shown that inhibition of HDAC3 activity can reduce the formation and fusion of osteoclasts, attenuate bone resorption, and promote bone healing under specific conditions (e.g., in female mice) [33].

KAT2B is a histone acetyltransferase (HAT), also known as P300/CBP-associated factor (PCAF). It mainly regulates gene transcriptional activity by acetylating specific sites such as lysine 9 of histone H3. Relevant studies have shown that PCAF (i.e., KAT2B) may promote osteoblast differentiation by acetylating Runx2 [34]. Previous studies have confirmed that p300/CBP can enhance the TGF-β/bone morphogenetic protein (BMP) signaling pathway by acetylating Smad proteins [35]. As a member of the HAT family, KAT2B is highly likely to share this mechanism, but its specific contribution to bone regeneration remains unknown.

SMARCA4, whose protein product is commonly known as Brahma-related gene 1, is a key catalytic subunit of the SWI/SNF (SWItch/Sucrose Non-Fermentable) chromatin remodeling complex. Studies have shown that SMARCA4 is essential for osteoblast differentiation induced by BMP-2. Brg1 can regulate the process of osteoblast differentiation by participating in the regulation of RUNX2 expression [36]. Furthermore, overexpression of SMARCA4 in mesenchymal stem cells (MSCs) can significantly upregulate the expression of a series of osteogenic marker genes, including RUNX2, Sp7, type I collagen, and osteopontin. This directly demonstrates that SMARCA4 possesses a strong ability to drive MSCs differentiation into the osteogenic lineage.

Tumor protein P53 (TP53) is an important transcription factor that regulates critical cellular processes, including cell cycle progression, DNA repair, apoptosis, and genomic stability [37]. Recent studies have found that functional defects or inactivation of the TP53 gene are closely associated with dysregulated osteogenic differentiation of MSCs. These molecular alterations drive a pathological imbalance in bone remodeling by promoting bone formation to exceed bone resorption. Disruption of the dynamic equilibrium between osteogenesis and osteoclastogenesis ultimately leads to abnormal skeletal homeostasis [38]. The dual effects of TP53 deficiency on the regulation of bone metabolism need to be comprehensively evaluated. Studies have reported that the loss of function of TP53 may enhance osteoblast differentiation, thereby increasing bone mineral density. This phenomenon has dual implications during fracture healing: accelerated osteogenic differentiation can shorten the healing timeline, whereas abnormally elevated bone density may impair microstructural reorganization of bone tissue, altering the mechanical properties of the repair site [39]. Additionally, TP53 is transiently suppressed during the initial phase of wound healing to promote proliferation and reactivated in later stages to facilitate tissue remodeling [40], highlighting the need to account for its spatiotemporal dynamics. Overall, TP53 orchestrates fracture healing through inflammatory responses and bone remodeling equilibrium across many phases of the healing process.

In this study, KEGG enrichment analysis revealed significant enrichment of pathways associated with the thyroid hormone signaling pathway and the Notch signaling pathway. Thyroid hormones (TH), primarily comprising thyroxine (T4) and triiodothyronine (T3), exert their effects via intracellular receptors to regulate diverse BP. The bone is a highly sensitive prototypical T3 target tissue that has been extensively studied for its key roles in skeletal development, growth, and adult bone remodeling and maintenance. For example, abnormal thyroid status is associated with a greater fracture risk in postmenopausal women [41]. TH treatment enhances insulin-like growth factor-1 expression in osteocytes and chondrocytes, promoting skeletal growth [42].

The Notch signaling pathway, a highly conserved intercellular communication mechanism, regulates cell fate determination, proliferation, differentiation, and apoptosis through direct cell-cell contact. Notch activation promotes the osteogenic differentiation of MSCs, but excessive activation may suppress mature osteoblast function. Studies have revealed upregulated expression of Notch pathway components (e.g., Jagged1 and NICD) in vascular endothelial cells of callus tissue [43]. Further investigations demonstrated the involvement of Notch in regulating type H vessel formation during bone repair [44]. Persistent Notch signaling activation in endothelial cells increases type H vessel abundance and enhances the proliferation of osteoprogenitor cells but decreases their differentiation into osteoblasts [45], thereby modulating the progression of fracture healing.

Through immune infiltration analysis, we highlighted the critical role of dysregulated immunomodulatory mechanisms in ATBC-induced impairments in the fracture healing process [46]. The differential infiltration of specific immune cell types between the five clusters highlights the importance of the immune microenvironment in skeletal health and disease. The role of immune cells in orchestrating the complex process of fracture healing has received substantial attention in the last few years. Studies have shown that within minutes of fracture occurrence, platelets and innate immune cells infiltrate the injury site, where they release proinflammatory cytokines and chemokines. During later stages, neutrophils and macrophages adopt an anti-inflammatory phenotype to clear cellular and tissue debris [47]. Consequently, the intricate interplay between bone cells and immune cells may influence bone density and architecture. Disruption of this equilibrium by external factors may lead to impaired fracture healing outcomes, thereby contributing to various fracture healing disorders.

The expression of the core target HDAC2 is significantly correlated with immune cells, such as Tregs and NK cells. Tregs at bone injury sites facilitate skeletal repair [48]. Treg levels at fracture sites increase about threefold compared to those at baseline, with Tregs secreting progranulin interacting with skeletal stem cells in a CCR8-CCL1-dependent manner, thereby driving fracture healing [49]. NK cells are important components of the innate immune system and recognize and induce the apoptosis or lysis of foreign, virus-infected, or metabolically altered cells post-tissue injury [50]. For example, NK cells rapidly infiltrate fracture sites post-injury, recruiting mesenchymal precursor cells via chemokines such as CXCL7 to promote repair [51]. The role of T cells and NK cells in fracture healing needs to be further investigated. TP53 immune-infiltration heatmaps highlight significant associations with neutrophils in this process. Neutrophils serve as the first line of immune surveillance and rapidly migrate to injury sites to initiate localized inflammatory responses and recruit other immune cells (e.g., macrophages) for pathogen clearance. In murine fracture models, neutrophil depletion impedes healing [52]. Studies have found that neutrophils polarize into distinct phenotypes during different healing phases: N1 pro-inflammatory neutrophils dominate the early inflammatory stage to recruit immune cells, whereas N2 anti-inflammatory neutrophils are recruited later, expressing pro-regenerative factors that promote bone regeneration [53]. Immune infiltration analysis highlights the risk of ATBC in disrupting fracture healing dynamics.

Non-coding RNAs play critical roles in fracture healing. Preclinical studies have identified miRNAs as key regulatory molecules in this process [54]. For example, hsa-miR-494-3p, a mature small RNA, promotes RANKL-induced osteoclastogenesis by targeting G protein-coupled receptor 4 in osteoclast precursors while simultaneously inhibiting osteoblast differentiation via SEMA3A suppression, suggesting that it may negatively regulate fracture healing [55]. In contrast, hsa-miR-218-5p, a multifunctional miRNA involved in bone metabolism and tumor suppression, enhances the osteogenic differentiation of BMSCs by targeting collagen type I alpha 1 [56]. To summarize, miRNAs exert significant regulatory effects across various stages of fracture healing, although their precise mechanisms of action require further investigation. Similarly, lncRNAs are indispensable in fracture repair. Metastasis-associated lung adenocarcinoma transcript 1 (MALAT1) is a highly conserved and abundant nuclear lncRNA that has been implicated in bone homeostasis. MALAT1 knockout mice exhibit marked osteoporotic bone phenotypes characterized by a decrease in osteoblast-mediated bone formation ability and enhanced osteoclast-driven bone resorption. MALAT1 binds β-catenin and modulates the β-catenin-OPG/Jagged1 pathway in osteoblasts and chondrocytes to promote bone formation and suppress bone resorption [57]. Nuclear paraspeckle assembly transcript 1 (NEAT1), another lncRNA involved in cell proliferation, differentiation, and genomic stability, regulates osteogenic gene expression by competitively binding miRNAs. In osteoporosis models, NEAT1 acts as a ceRNA to sequester miR-466f-3p, thereby upregulating hexokinase 2 expression, inhibiting osteoblast autophagy, and impairing bone formation [58]. Conversely, knocking down NEAT1 suppresses the osteogenic differentiation of human BMSCs [59]. We predicted and validated 3 miRNAs and 16 lncRNAs linked to two core targets. An mRNA-miRNA-lncRNA regulatory network was constructed, revealing intricate interactions among these molecules. This network highlights important mechanisms by which ATBC may disrupt fracture healing, warranting further investigation.

This study confirmed the crucial role of core targets in ATBC’s impact on fracture healing through molecular docking results. To further validate stability, we performed molecular dynamics simulations and systematically calculated the following metrics: RMSD, RMSF, Rg, SASA, MM/GBSA, and FEL. Notably, interacting residues were highlighted in the decomposition analysis of MD simulation results, demonstrating strong agreement between our molecular docking and dynamics outcomes. These findings indicate significant binding activity and robust stability between five core targets and ATBC. From a molecular mechanism perspective, this enhanced stability may prolong the in vivo residence time of the complex, thereby continuously modulating target protein function and influencing fracture healing progression. This provides key theoretical insights for elucidating ATBC’s molecular pathological mechanism in fracture healing.

### Limitations

This study also has certain limitations. First, the risk of ATBC in impairing fracture healing was not validated experimentally. Second, the sample size of the dataset used in this study is relatively small, which may introduce bias into the results; meanwhile, we urge relevant researchers to provide more high-quality datasets related to fracture healing. Third, the outcomes could be influenced by algorithmic biases, source heterogeneity, or data quality issues. Future studies need to focus on standardized in vitro and in vivo experiments supplemented by in-depth bioinformatics analysis to validate core targets and signaling pathways identified via network toxicology analyses, along with long-term, multicenter, high-quality epidemiological studies to monitor and evaluate the evolving relationship between exposure to ATBC and fracture healing outcomes.

## Conclusion

This study systematically investigated the relationship between ATBC and fracture healing for the first time using network toxicology, bioinformatics, molecular docking, and molecular dynamics simulation methods. To some extent, these findings provided novel information and perspectives for the clinical diagnosis and treatment of fracture nonunion, offering a framework to better understand the interplay between environmental toxicants and skeletal repair.

## Data Availability

The datasets generated during and/or analysed during the current study are available from the corresponding author on reasonable request.

## Abbreviations

ATBC: Acetyl tributyl citrate
DEGs: Differentially expressed genes
PPI: Protein-protein interaction
GO: Gene Ontology
KEGG: Kyoto Encyclopedia of Genes and Genomes
BP: Biological process
CC: Cellular component
MF: Molecular function
ssGSEA: Single-sample gene set enrichment analysis
GSEA: Gene set enrichment analysis
ceRNA: Competing endogenous RNA
RESP: Restrained electrostatic potential
RMSD: Root mean square deviation
RMSF: Root mean square fluctuation
Rg: Radius of gyration
SASA: Solvent accessible surface area
MM/GBSA: Molecular mechanics/generalized Born surface area
FEL: Free energy landscape
NK: Natural killer
Tregs: Regulatory T cells
PCA: Principal component analysis
OPN: Osteopontin
BSP: Bone sialoprotein
OC: Osteocalcin
BMSCs: Bone marrow mesenchymal stem cells
HAT: Histone acetyltransferase
PCAF: P300/CBP-associated factor
BMP: Bone morphogenetic protein
MSCs: Mesenchymal stem cells
TH: Thyroid hormones
T4: Thyroxine
T3: Triiodothyronine
MALAT1: Metastasis-associated lung adenocarcinoma transcript 1
NEAT1: Nuclear paraspeckle assembly transcript 1
